# Evaluation of Yeast Hydrolysate in a Low-Fishmeal Diet for Whiteleg Shrimp (*Litopenaeus vannamei*)

**DOI:** 10.3390/ani13111877

**Published:** 2023-06-05

**Authors:** Ali Hamidoghli, Yein Lee, Soyeon Hwang, Wonsuk Choi, Youn-Hee Choi, Sungchul C. Bai

**Affiliations:** 1 Feeds & Foods Nutrition Research Center, Pukyong National University, Busan 48513, Republic of Korea; 2 Aquaculture Research Institute, University of Idaho, Hagerman, ID 83332, USA; 3 Department of Fisheries Biology, Pukyong National University, Busan 48513, Republic of Korea; 4 Division of Fisheries Life Sciences, Pukyong National University, Busan 48513, Republic of Korea

**Keywords:** alternative protein, sustainable aquaculture, reactive oxygen species, growth, histology

## Abstract

**Simple Summary:**

Strategies to reduce the amount of marine ingredients in the diet of commercially important species such as whiteleg shrimp have received great attention. Yeast products obtained from *Saccharomyces cerevisiae* are a rich source of protein, amino acids and energy. Furthermore, yeast is a abundant source of nucleotides, beta-glucans, mannan oligosaccharides, chitin and peptides that have shown to improve immune response, growth performance and stress resistance in shrimp. The results of the present study showed that supplementing 4% of hydrolyzed yeast in shrimp diet with low fishmeal (10%) could improve growth, intestinal morphology and disease resistance.

**Abstract:**

An eight-week feeding trial was performed to evaluate the effects of yeast hydrolysate (YH) supplementation in a low-fishmeal diet on the growth, immune responses, intestinal histology and disease resistance of whiteleg shrimp (*Litopenaeus vannamei*). Five experimental diets were produced by supplementing YH at 0 (CON), 0.5 (YH_0.5_), 1 (YH_1_), 2 (YH_2_) and 4 (YH_4_) % to a basal diet containing 10% fishmeal and compared with a positive control with 25% fishmeal (FM_25_). Shrimp with an initial average weight of 0.43 ± 0.005 g (mean ± SD) were stocked in 18 tanks and fed the experimental diets (38% protein and 8% lipid) four times a day. Results showed that shrimp fed the FM_25_ diet exhibited significantly higher final body weight, weight gain, specific growth rate and protein efficiency ratio than those fed CON, YH_0.5_, YH_1_ and YH_2_ diets (*p* < 0.05). However, there were no significant differences between shrimp fed the YH_4_ and FM_25_ diets (*p* > 0.05). In addition, there were no significant differences in whole-body proximate composition, hemolymph biochemical parameters and non-specific immune responses among treatments. Intestinal villi length and muscular layer thickness of shrimp fed the YH_4_ and FM_25_ diets were significantly higher than the other groups. At the end of the bacterial (*Vibrio parahaemolyticus*) challenge test, shrimp fed YH_4_ and FM_25_ diets showed a significantly higher survival rate than those of shrimp fed CON, YH_0.5_ and YH_1_ (*p* < 0.05). These results suggest that supplementing 4% YH in diet containing 10% fishmeal could beneficially influence growth, intestinal morphology and disease resistance of whiteleg shrimp.

## 1. Introduction

Whiteleg shrimp, *Litopenaeus vannamei*, is probably the most lucrative species in aqua culture, with 5.8 million metric tons of production in 2020 accounting for a value of approximately USD 34 billion [1]. To satisfy the global market demands for this species, sustainable development of shrimp aquaculture industry is highly required. Challenges such as excessive use of marine ingredients (e.g., fishmeal and fish oil) in diet, high mortality due to improper nutrition and excessive feed prices are major impediments in shrimp aquaculture [2,3].

Fishmeal and fish oil are among the best protein and lipid sources in shrimp diets. Fishmeal contains sufficient amounts of essential amino acids and possesses high digestibility and palatability in shrimp nutrition [4,5,6,7]. However, fishmeal is not sustainably sourced, not available in required quantities and not cheap. To meet the growing demands for shrimp feed, several researchers have examined the use of alternative protein sources in shrimp diet production. These researchers reported the successful use of ingredients originating from plant [8], animal [9,10] and microbial sources [11] as alternatives or complements of fishmeal in shrimp diets. Microbial protein is obtained from different microbial biomass sources such as yeast bacteria and microalgae, with great potential to partially or even completely replace fishmeal in shrimp diets [12].

Yeast products obtained from *Saccharomyces cerevisiae* (brewer’s yeast) have been used in animal and human diets for decades [13,14]. The rich protein, amino acid and energy content of yeast makes it a suitable ingredient for aquatic species including whiteleg shrimp [15,16,17]. Furthermore, yeast is a rich source of nucleotides, beta-glucans, mannan oligosaccharides, chitin and peptides [12,18]. These compounds have shown to improve immune response, growth performance and stress resistance in whiteleg shrimp [19,20,21,22,23]. However, the yeast cell wall is not easily digested by aquatic animals and low digestibility values are often observed when unprocessed yeast is used in shrimp diets [17,24]. Low digestibility can result in poor utilization of yeast nutrients and active compounds by the animal. Another important factor is the price of yeast products after processing. Practical incorporation of yeast products in shrimp diet requires thorough consideration of economic feasibility. With a price almost identical to fishmeal, yeast hydrolysate improved the growth performance and feed utilization of whiteleg shrimp fed a diet with 20% fishmeal [22]. Moreover, improved defense against low salinity stress by modulating superoxide dismutase, alkaline phosphatase and acid phosphatase activities was observed in shrimp fed 3% yeast hydrolysate [23]. Nevertheless, a dose-dependent response of whiteleg shrimp to dietary yeast hydrolysate in low-fishmeal diets has not been investigated. Therefore, based on previous literature, the present study aimed at evaluating the effects of graded levels of yeast hydrolysate in low-fishmeal diets (10%) on growth, body composition, non-specific immune responses, hematological parameters, intestinal morphology and disease resistance in juvenile whiteleg shrimp *L. vannamei*.

## 2. Materials and Methods

### 2.1. Experimental Design and Diets

The commercially available yeast hydrolysate (YH, *Saccharomyces cerevisiae*) was provided in dry powder by ANGEL YEAST Co., Ltd. (Yichang, Hubei, China). *S. cerevisiae* by-products originate from the food industry and YH is processed through enzyme-mediated hydrolysis as previously described [16]. The proximate composition and the amino acid content of YH were analyzed at the Feeds & Foods Nutrition Research Center (FFNRC, Busan, Republic of Korea, Table 1). Fishmeal (Anchovy), fermented soybean meal, soybean meal, corn gluten, squid liver powder, poultry by-product and meat and bone meal were used as protein sources for the six isonitrogenous (~38% crude protein) and isolipidic (~8% crude fat) diets (Table 2). YH was added at 0 (CON), 0.5 (YH_0.5_), 1 (YH_1_), 2 (YH_2_) and 4 (YH_4_)% to five experimental diets containing 10% fish meal. A positive control with 25% fishmeal (similar to a commercial shrimp diet) was also used in this experiment. After mixing the experimental raw materials, distilled water and fish oil were added to the dry feed and mixed thoroughly using an electric mixer (HYVM-1214, Hanyoung Food Machinery, Hanam, Republic of Korea). Then, a laboratory scale pelleting machine (SFDGT, Shinsung, Republic of Korea) was used to produce the pellet feed. Diets were air-dried for 24 h until the moisture content was less than 10%, and then stored at −20 °C.

### 2.2. Experimental Shrimp and Feeding Trial

Procedures for rearing and nutrition of whiteleg shrimp were followed as previously described [3,5]. Whiteleg shrimp (0.2 ± 0.05 g) were purchased from a shrimp farm (Palddak shrimp) located in Goseong, Gyeongsangnam-do, Republic of Korea and moved to the Feeds & Foods Nutrition Research Center (FFNRC), Busan, Republic of Korea. The experimental shrimp were acclimatized to the experimental conditions in 200 L tanks for three weeks while a commercial feed was used (Suhyup Feed Co., Uiryeong-eup, Republic of Korea) to feed them four times a day (40% protein). Water was exchanged daily by draining up to 30%. To begin the trial, 20 whiteleg shrimp with an average weight of 0.43 ± 0.005 g (mean ± SD) were randomly distributed in 18 rectangular tanks with 40 L capacity. Three tanks were assigned to each experimental diet and feeding was performed for a total of eight weeks. During the experiment, the water temperature was maintained at 28.0 ± 0.2 °C using a water tank heater, the dissolved oxygen was maintained at 5.7 ± 0.1 mg/L using air stones connected to an air pump (HP-200, HIBLOW, Incheon, Republic of Korea), the pH was 7.77 ± 0.05 and salinity was 27–30 ppt. Shrimp were reared in a semi-recirculating system comprising of rearing tanks that receive water at 1.2 L/min and a reservoir tank with physical and coral reef filtrations. Dark plastic covers were used on top of all the experimental tanks to reduce stress. Shrimp were fed their respective diets four times a day (9:00, 12:30, 15:30 and 19:00) at apparent satiation and the feed supply was adjusted according to the growth and mortality. Siphoning was performed twice a day to remove feces. All tanks were cleaned by scrubbing the walls and 20% of the total seawater was replaced daily.

### 2.3. Sample Collection and Analysis

#### 2.3.1. Growth Performance

At the beginning and end of the experiment, the total number and weight of the experimental shrimp were measured after fasting for 12 h. Shrimp initial body weight (IBW), final body weight (FBW), weight gain (WG), specific growth rate (SGR), feed efficiency (FE), protein efficiency ratio (PER) and survival (SR) were calculated according to the formulas described by Bai et al. [25]:

Weight gain (WG, %) = [final weight (g) − initial weight (g)]/initial weight (g) × 100

Specific growth rate (SGR, %/day) = [In final weight (g) − In initial weight (g)]/days × 100

Feed efficiency (FE, %) = (wet weight gain/dry feed intake) × 100

Protein efficiency ratio (PER) = wet weight gain (g)/protein intake (g)

Survival rate (SR, %) = (final number of shrimp/initial number of shrimp) × 100

#### 2.3.2. Proximate Composition Analysis

Six shrimp from each tank (with shells) were ground, pooled and stored at −20 °C for whole-body composition analysis. Whole-body and feed samples were analyzed according to the AOAC (Association of Official Analytical Chemists, 2005) method [26]. Ash content was analyzed using a sample of constant weight and burning it in a muffle furnace at 550 °C for 3 h (DAIHAN, WiseTherm^®^, Kangwon-do, Republic of Korea). The moisture was analyzed using an atmospheric pressure heating and drying method in which samples with a certain weight were heated in a dry oven (OF02G-4C, WiseVen^®^, Wertheim, Germany) at 135 °C for 3 h. In order to analyze the crude fat and crude protein of whole shrimp, the samples were freeze-dried (Advantage 2.0, VirTis, New York, NY, USA) and then prepared in powder form. Thereafter, crude protein was analyzed using Kjeldahl nitrogen quantification (Nitrogen × 6.25) method (Buchi B324/435/412, Flawil, Switzerland; Metrohm 8-719/806, Flawil, Switzerland). Crude fat was analyzed using the Soxhlet extraction method using the Soxtec system 1046 (Tecator AB, Höganäs, Sweden).

#### 2.3.3. Hematological Analysis

After the feeding trial, six shrimp were randomly taken out from each tank, and approximately 0.3 mL of hemolymph was collected from the abdominal cavity (the first abdominal portion) using a 1 mL syringe. The hemolymph was transferred to a 1.5 mL microtube and left at room temperature for 30 min. After clotting, hemolymph samples were centrifuged at 12,000 rpm 4 °C for 10 min using a centrifuge (Hanil Scientific Microcentrifuges M15R, Gimpo, Republic of Korea) to separate the serum. Thereafter, the separated serum was stored at −20°C until use for hematological analysis and non-specific immunoassays. Glucose, aspartate aminotransferase (AST) and alanine aminotransferase (ALT) were analyzed with DRI-CHEM 4000i (Minato-ku, Tokyo, Japan) using a FujiFilm Dri Chem kit.

#### 2.3.4. Non-Specific Immune Response Analysis

Superoxide dismutase (SOD) was measured using a SOD Assay Kit (Dojindo Laboratories, Kumamoto, Japan) and following the manufacturer’s instructions. This kit measures SOD activity in the sample using water soluble tetrazolium salt (WST). First, the inhibition rate of enzymes using WST-1 and xanthine oxide was calculated as a percentage. Then, each sample was incubated at 37 °C for 20 min and the absorbance was measured at 450 nm wavelength using a microplate reader (AMR-100 Microplate ELISA Reader). The SOD activity was expressed as percent inhibition and one unit of SOD was defined as the amount of enzyme capable of inhibiting the reduction reaction of dye and O_2_^−^ by 50%.

The lysozyme activity analysis was performed based on the method of Ellis [27]. *Micrococcus lithodeikticus* (Sigma) was mixed with sodium citrate buffer (0.02 M, pH 5.52) to prepare a bacterial suspension (0.2 mg/mL). Serum dilution was prepared by mixing 30 μL of serum and 270 μL of sodium citrate buffer (0.02 M, pH 5.52). A 180 μL bacterial suspension was added to the 96-well plate and 20 μL serum dilution was added to react. This was incubated at room temperature (20 °C) and, after reaction, the absorbance of the sample was measured at a wavelength of 450 nm at 0, 30, and 60 min in a microplate reader (AMR-100 Microplate ELISA Reader). The active unit of lysozyme was defined as the amount of enzyme indicating a decrease in absorbance of 0.001 per minute.

#### 2.3.5. Histomorphology of the Intestine

Two shrimp were separated from each tank and used for the histomorphological analysis. A 1 cm section of the mid-intestine was removed from each shrimp and fixed in 10% neutral formaldehyde. Intestine sections were dehydrated using ascending ethanol series and embedded with paraffin. Next, the paraffin blocks were sliced at 5 μm thickness using a microtome (CUT 4055, MicroTec, Walldorf, Germany) and each section was attached to a glass slide (six slides per tank), dried on a heating plate (45 °C) and stained with hematoxylin and eosin (H&E). After staining, slides were sealed using Canada Balsam, followed by observation using a microscope (Olympus BX41, Tokyo, Japan) equipped with a digital camera for microscopy (DIXI Optics, Daejeon, Republic of Korea). Images were taken through Mosaic 2 image analysis software (Mosaic Laboratories, LLC., Lake Forest, CA, USA). Using this software, the villus length (VL) and the muscular layer thickness (MLT) of the shrimp intestines were evaluated. VL (μm) corresponds to the length of villi from the top to the bottom and MLT (μm) refers to the distance from the circular muscle layer to the intestinal epithelial layer. For each sample, 10 measurements were performed per section to obtain an average value.

### 2.4. Challenge Test

After eight weeks of the feeding trial, seven shrimp were selected from each tank and used in the bacterial challenge test against *Vibrio parahaemolyticus*. Three repetitions were considered for each treatment (total of 18 tanks). The pathogenic bacteria *V. parahaemolyticus* used in this experiment was obtained from infected shrimp by the Institute of Microbiology at Dong-A University, Republic of Korea. Pathogenic bacteria were incubated in a 10 mL brain heart infusion broth and incubated at 37 °C for 24 h in a shudder. Bacterial growth was observed with a spectrophotometer at 600 nm light density. Then, bacteria were collected via centrifugation and washed twice with phosphate-buffered saline (0.1 M, pH 7.0). As previously described [28], phosphate-buffered saline containing *V. parahaemolyticus* cells was adjusted to 1.75 × 10^6^ CFU/mL, and 25 μL of this suspension was injected into the abdominal cavity of the shrimp. During the challenge test, the shrimp were not fed, the water temperature was maintained at 28–29 °C and the mortality rate was recorded for 84 h to calculate the cumulative survival rate.

### 2.5. Statistical Analysis

The results of this experiment were presented as mean ± standard deviation (SD). The statistical analysis results of this experiment were processed using SPSS 27.0 (Statistical Package for Social Science, SPSS Inc., Chicago, IL, USA) software. After checking the homogeneity and normality of data, a one-way ANOVA test was used to show difference among all treatments (*p* < 0.05). When a significant difference was observed, Duncan’s multiple range test was used to compare different treatments.

## 3. Results

### 3.1. Growth Performance

The final body weight (FBW), weight gain (WG), specific growth rate (SGR) and protein efficiency ratio (PER) of shrimp fed FM_25_ were significantly higher than those of shrimp fed CON, YH_0.5_, YH_1_ and YH_2_ diets (*p* < 0.05, Table 3). However, there were no significant differences between shrimp fed YH_4_ and FM_25_ diets (*p* > 0.05). The feed efficiency of shrimp fed FM_25_ was significantly higher than those of shrimp fed all the other diets. Although there were no significant dereferences in survival percentage of shrimp fed all the experimental diets (*p* > 0.05), shrimp fed the FM_25_ diet showed a slightly higher survival.

### 3.2. Whole Body Proximate Composition

No significant difference in whole body proximate composition was found among all treatments (*p* > 0.05, Table 4).

### 3.3. Hematological Analysis

The amounts of aspartate aminotransferase (AST) and alanine aminotransferase (ALT) enzymes, as well as serum glucose (GLU) contents, were not significantly influenced by different dietary treatments (*p* > 0.05, Table 5).

### 3.4. Non-Specific Immune Response Analysis

There were no significant differences in superoxide dismutase (SOD) and lysozyme activity of shrimp fed all the experimental diets (*p* > 0.05, Table 6).

### 3.5. Histomorphology of the Intestine

**Figure 1:**
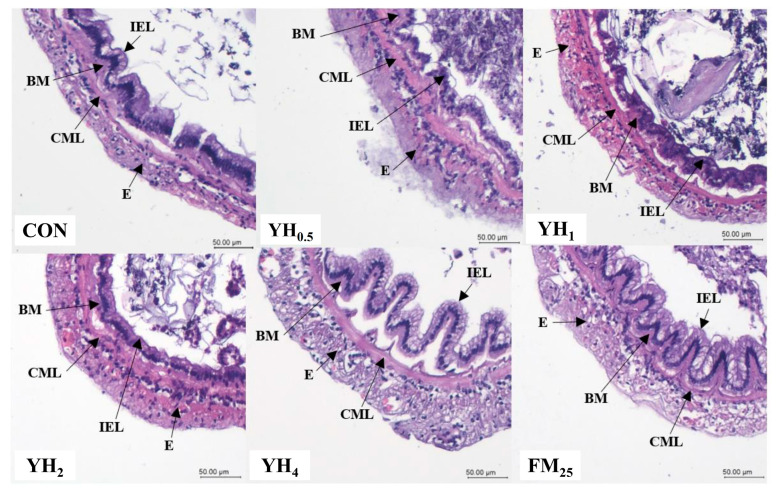
Histological structure with hematoxylin and eosin staining of the intestine of whiteleg shrimp fed the experimental diets for 8 weeks. E, external muscular layer; BM, basement membrane; CML, circular muscle layer; IEL, intestinal epithelial layer. Image is ×100 (scale bar, 50 μm). For diet information refer to Table 2.

The arrangement of the intestinal villi of shrimp fed YH_4_ and FM_25_ diets was more orderly and showed more tidiness than other treatments (Table 7). In addition, the villi length and muscular layer thickness of the mid-intestine for the shrimp fed YH_4_ and FM_25_ diets were significantly higher than other treatments (*p* < 0.05). However, shrimp fed FM_25_ had significantly higher muscular layer thickness compared to those fed the YH_4_ diet (Figure 1).

### 3.6. Challenge Test

**Figure 2:**
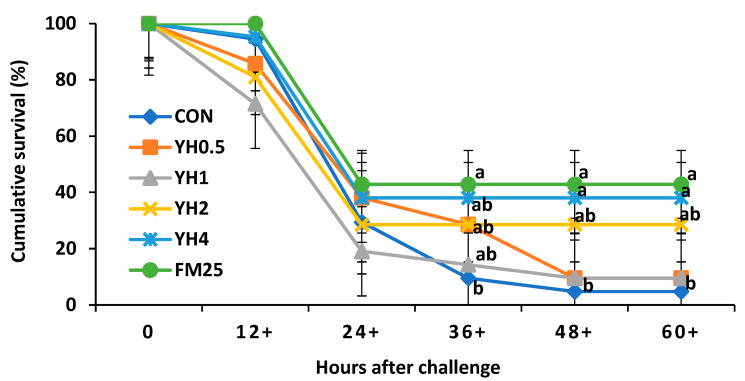
Cumulative survival (%) of whiteleg shrimp fed different dietary levels of yeast hydrolysate (YH) and injected with *Vibrio parahaemolyticus* (25 μL at 1.75 × 10^6^ CFU/mL). Values at each time point with different superscripts are significantly different (*p* < 0.05). For diet information refer to Table 2.

During the challenge test, the first mortalities occurred at 12 h, with no significant differences among dietary treatments (Figure 2). At 36 h after the challenge test, shrimp fed FM_25_ showed a significantly higher cumulative survival than those fed the CON diet (*p* < 0.05). However, there were no significant differences in cumulative survival of shrimp fed the FM_25_, YH_0.5_, YH_1_, YH_2_, and YH_4_ diets. At 48 h after the challenge, shrimp fed FM_25_ and YH_4_ showed a significantly higher cumulative survival than those fed CON, YH_0.5_, and YH_1_ diets. There were no significant differences in survival of shrimp fed FM_25_, YH_4_, and YH_2_ at 48 h after the challenge (*p* > 0.05). Results were stagnant at 60 h after the challenge test.

## 4. Discussion

In the present experiment, the positive control diet was 25% fishmeal, which is comparable to shrimp commercial diets [15,22]. Other experimental diets consisted of 10% fishmeal which is considered “low” in Asian shrimp aquaculture. Previous studies have shown the great potential of yeast products in low-fishmeal diets for whiteleg shrimp [15,17,18,24]. Additionally, the positive effects of yeast hydrolysate on growth, immune responses, antioxidant capacity, stress resistance and gene expression of whiteleg shrimp was proven in previous studies [22,23]. However, to the best of our knowledge, a dose-dependent response of dietary yeast hydrolysate in low-fishmeal diets for whiteleg shrimp has not been investigated.

In the present experiment, the FBW, WG, SGR and PER of shrimp fed the positive control diet (25% fishmeal) were higher than all other diets, except for the diet with 4% yeast hydrolysate. This shows that the highest level (4%) of yeast hydrolysate could have compensated for the nutritional insufficiencies in the low-fishmeal diets. In a study, it was shown that fishmeal can be reduced from 25% to 20% in whiteleg shrimp diet when 3% of yeast extract is used [22]. A straightforward explanation for these observations could be the rich nutritional composition of yeast products, as was shown in several previous studies [29]. Yeast products, especially those obtained from *Saccharomyces cerevisiae*, have a relatively high protein content, balanced amino acid composition and a rich amount of nucleotide [29]. Nucleotides are intracellular compounds that build up nucleic acids and have key roles in a wide range of biochemical processes [30]. Fishmeal itself is a rich source of nucleotides and replacement of fishmeal may result in a significant drop in nucleotides, as well as essential amino acids [31]. Although nucleotides are not essential nutrients, they play critical roles in feeding attraction and stimulation, growth promotion, immunostimulantion, stress responses and disease resistance [32]. It was reported that dietary nucleotides (0.9%) enhanced immune response, gastrointestinal health and disease resistance in whiteleg shrimp fed a low-fishmeal diet [33]. The yeast hydrolysate used in our experiment contains 12.17% of nucleic acids [16], and this can partly explain our growth enhancement results. Another explanation could be related to the high digestibility of yeast products in shrimp diet. Yeast products have a relatively high digestibility in shrimp diet that could be related to the quality of the protein [15,17]. Nevertheless, further studies with higher levels of yeast hydrolysate in shrimp diet are required to observe linear additive effects.

Shrimp whole-body proximate composition was not significantly affected as a response to different dietary treatments in this study. Similar to our results, differences in body protein content of whiteleg shrimp when fed different levels of yeast and yeast extract were not observed [18]. However, an increase in body crude lipid content with higher yeast supplementations was reported [18], which contradicts our results. In our study, the whole-body crude lipid content of shrimp fed increasing levels of yeast hydrolysate showed a slight increase compared to the negative control group, but differences were not significant. This might be due to the fact that shrimp muscle contains low levels of fat compared to fish [34], and therefore differences might be negligible. Similar to the body composition results, the hemolymph biochemical parameters, such as aspartate aminotransferase (AST), alanine aminotransferase (ALT) and glucose, were not affected by dietary yeast hydrolysate and/or low fishmeal. AST and ALT are enzymes that are used as indicators of liver health and function [35] and serum glucose is an indicator of stress in aquatic animals [36]. A previous study has shown that suboptimal conditions such as induction of hypothermal stress have resulted in increased glucose levels in shrimp hemolymph [37]. On the contrary, optimum levels of dietary yeast nucleotides can reduce the glucose levels in fish after acute stress [38]. The stagnant AST, ALT and glucose levels in our experiment might be because the nutrient insufficiencies or experimental conditions were not severe enough to damage the shrimp hepatopancreas. Future studies are needed with a focus on the effects of yeast hydrolysate on hemolymph parameters in shrimp while a stress challenge is induced.

Superoxide dismutase (SOD) enzyme is the part of the antioxidant defense mechanisms that scavenges the superoxide anion (O_2_^−^), and lysozyme is an antimicrobial enzyme that can lyse cell membranes of pathogenic bacteria. SOD and lysozyme are two major indicators of innate immune responses [39,40]. Our results showed no significant differences in SOD and lysozyme activities of shrimp hemolymph fed different levels of yeast hydrolysate in low-fishmeal diets. Similar to our results, no changes were observed in SOD activity when 3% of yeast extract was used to replace concentrated cottonseed protein in whiteleg shrimp [18]. In contrast, in another study, it was reported that dietary yeast hydrolysate increased the SOD activity of shrimp when salinity stress was induced [23]. It seems that the effects of dietary yeast products on immune responses are more obvious when extreme challenges are exposed. In fish fed graded levels of yeast hydrolysate, it was notably shown that plasma lysozyme, SOD and IgM were only significant after the pathogenic challenge test [16]; these parameters were not significantly affected by dietary yeast hydrolysate before the challenge test. This might indicate that the positive effects of dietary yeast hydrolysate are more visible when fish/shrimp are challenged or stressed.

The intestinal morphology of aquatic organisms directly reflects the quality of feed and incorporated ingredients. For example, high levels of unprocessed soybean meal is known to induce intestinal inflammation in fish [41]. Moreover, intestinal morphological status such as villi length, muscular layer thickness and number of goblet cells play a significant role in digestion and absorption of nutrients [42]. The results of the present experiment have shown a clear and significant increase in intestinal villi length and muscular layer thickness when 4% yeast hydrolysate was used in shrimp diet. This can partly explain our observations in growth performance parameters. In line with our findings, it was reported that 1.5% dietary baker’s yeast extract improved the structure of hepatocytes and villi in fish [43]. It was also shown that dietary nucleotides, glucans and mannan oligosaccharides, which are relatively abundant in yeast hydrolysate, enhance intestinal morphometric characteristics [30,44]. Other than this, we observed a decrease in villi length in group YH_2_ compared to YH_1_ that does not seem to have a clear explanation, and further studies in this regard are required.

Whiteleg shrimp fed the positive control and 4% yeast hydrolysate showed the highest cumulative survival after the challenge with *Vibrio parahaemolyticus*, but with no significant difference with the YH_2_ group. Vibriosis is caused by *Vibrio parahaemolyticus*, which is a common disease in shrimp farms leading to high mortality and substantial economic losses [44]. One of the greatest challenges in shrimp farming is the prevention and control of disease outbreaks. Shrimp, unlike teleost fish, lack adaptive immune mechanisms and this highlights the importance of innate immunity [45,46]. Yeast and its derivatives have previously been shown to improve the innate immune responses of shrimp, leading to higher survival against *Vibrio parahaemolyticus* [47,48,49]. This could be explained by the presence of compounds such as ß–glucan and nucleotides in yeast. These compounds are well-known for their stimulatory effects on the innate immune responses of aquatic animals [30,50]. Nevertheless, as mentioned before, our results on lysozyme and SOD activity did not show significant differences as a response to dietary yeast hydrolysate. Moreover, we did not observe the same trends in shrimp survival percentage during the growth trial. It could be possible that the slight (non-significant) differences in shrimp survival during the growth trial were caused by non-nutritional (environmental) factors. Additionally, according to a previous study [16] and based on the results of the present experiment, it seems that the positive effects of yeast products are more obvious at suboptimal conditions and/or after bacterial challenge. Further studies are required on survival and the changes in innate immune responses of shrimp after bacterial challenge tests.

## 5. Conclusions

In conclusion, the present study showed that the inclusion of 4% yeast hydrolysate was beneficial in low-fishmeal diets for whiteleg shrimp. Our results indicated that the inclusion of 4% yeast extract in a low-fishmeal diet for whiteleg shrimp could have beneficial effects on growth, intestinal morphology and disease resistance. In addition, supplementation of low-fishmeal diets with 4% yeast extract could be comparable to those of high-fishmeal (25%) diets in whiteleg shrimp, *Litopenaeus vannamei*.

## Figures and Tables

**Table 1:** Proximate composition and amino acid analysis of yeast hydrolysate (YH).

Proximate Composition (% of Dry Matter (DM) Basis)	
Protein	53.06
Fat	0.31
Ash	6.52
Moisture	8.76
Amino acid analysis (% of DM basis)	
Alanine	4.86
Arginine	2.53
Aspartic acid	5.26
Cysteine	0.62
Glycine	2.33
Glutamic acid	7.31
Histidine	1.73
Isoleucine	2.47
Leucine	3.26
Lysine	3.51
Methionine	0.27
Phenylalanine	2.14
Proline	3.01
Serine	2.29
Threonine	2.34
Tyrosine	1.68
Valine	2.82

**Table 2:** Composition of the six experimental diets supplemented with yeast hydrolysate (YH) at different levels (% of DM basis).

Ingredients	Experimental Diets ^1^					
	CON	YH_0.5_	YH_1_	YH_2_	YH_4_	FM_25_
Fish meal (anchovy) ^2^	10.00	10.00	10.00	10.00	10.00	25.00
Fermented soybean meal ^3^	10.00	10.00	10.00	10.00	10.00	10.00
Soybean meal ^2^	14.90	14.80	14.60	14.00	13.10	11.90
Corn gluten ^2^	4.00	4.00	4.00	4.00	3.30	1.80
Squid liver powder ^2^	3.90	3.90	4.20	4.60	4.70	4.20
Wheat flour ^4^	20.00	20.00	20.00	20.00	19.88	22.00
Poultry by product ^2^	8.00	8.00	8.00	8.00	8.30	1.30
Meat and bone meal ^2^	7.00	6.70	6.20	5.70	5.40	1.90
Corn starch ^2^	12.88	12.78	12.68	12.38	12.00	13.28
Yeast hydrolysate ^5^	0.00	0.50	1.00	2.00	4.00	0.00
Lysine ^6^	0.90	0.90	0.90	0.90	0.90	0.50
Methionine ^6^	0.40	0.40	0.40	0.40	0.40	0.30
Vitamin premix ^7^	2.00	2.00	2.00	2.00	2.00	2.00
Mineral premix ^8^	2.00	2.00	2.00	2.00	2.00	2.00
Cholesterol ^9^	0.02	0.02	0.02	0.02	0.02	0.02
Fish oil ^2^	4.00	4.00	4.00	4.00	4.00	3.80
Total	100.00	100.00	100.00	100.00	100.00	100.00
Proximate composition (% of DM basis)						
Crude protein	37.78	38.56	38.82	38.43	38.27	38.74
Crude fat	8.46	8.49	8.35	8.37	8.39	8.41
Crude ash	8.50	8.51	8.46	8.48	8.50	8.45
Moisture	10.09	9.81	9.97	9.95	9.97	9.77

^1^ Experimental diets were regarded as a control (without YH) and 4 other diets containing YH: YH_0.5_ = Yeast hydrolysate at 5 g/kg; YH_1_ = YH at 10 g/kg; YH_2_ = YH at 20 g/kg; YH_4_ = YH at 40 g/kg; Additionally, a positive control diet containing 25% fishmeal (FM_25_). ^2^ The Feed Co., Goyang, Republic of Korea. ^3^ CJ Co., Seoul, Republic of Korea. ^4^ Samhwa FlourMils Co., Seoul, Republic of Korea. ^5^ ANGEL YEAST Co., Ltd. (Yichang, Hubei, China). ^6^ Sigma-Aldrich Korea Yongin, Republic of Korea. ^7^ Vitamin premix (mg/kg diet): A, 20,000 IU; D, 4000 IU; E, 200; B1, 40; B6, 30; B12, 0.2; C, 200; Calcium pantotenic acid, 100; Nicotinic acid 90; B-Biotin 0.2; Choline chloride, 600; Inositol, 100. ^8^ Mineral premix (g/kg diet): Ferrous fumarate, 0.25; Manganese sulfate, 0.225; Dried ferrous sulfate, 0.4; Dried cupric sulfate, 0.025; Cobaltous sulfate, 0.015; Zinc sulfate KVP, 0.275; Cancium iodate, 0.015; Magnesium sulfate, 1.604; Aluminum Hydroxide, 0.015. ^9^ Fujifilm Wako Pure Chemical Corporation, Hong Kong, China.

**Table 3:** Growth performance and survival of whiteleg shrimp fed the six experimental diets for eight weeks (Mean ± SD of triplicate observations) ^1^.

Parameters ^2^	Diets						*p*-Value
	CON	YH_0.5_	YH_1_	YH_2_	YH_4_	FM_25_	
IBW	0.43 ± 0.00	0.43 ± 0.00	0.43 ± 0.00	0.43 ± 0.01	0.44 ± 0.00	0.44 ± 0.00	0.276
FBW	2.29 ± 0.13 ^b^	2.28 ± 0.15 ^b^	2.45 ± 0.27 ^b^	2.33 ± 0.13 ^b^	2.58 ± 0.05 ^ab^	2.87 ± 0.08 ^a^	0.016
WG	434.27 ± 33.25 ^b^	426.70 ± 30.61 ^b^	464.70 ± 59.43 ^b^	439.17 ± 23.86 ^b^	492.08 ± 4.89 ^ab^	554.21 ± 18.31 ^a^	0.018
SGR	3.49 ± 0.13 ^b^	3.46 ± 0.12 ^b^	3.59 ± 0.23 ^b^	3.51 ± 0.09 ^b^	3.71 ± 0.02 ^ab^	3.91 ± 0.06 ^a^	0.029
FE	36.56 ± 3.55 ^b^	34.39 ± 0.77 ^b^	36.57 ± 2.97 ^b^	35.17 ± 1.65 ^b^	38.78 ± 4.58 ^b^	46.53 ± 0.76 ^a^	0.010
PER	1.00 ± 0.09 ^b^	0.94 ± 0.03 ^b^	0.98 ± 0.08 ^b^	0.97 ± 0.03 ^b^	1.05 ± 0.09 ^ab^	1.19 ± 0.02 ^a^	0.022
SR	90.00 ± 4.08	81.67 ± 4.71	80.00 ± 7.07	78.33 ± 4.71	83.33 ± 10.27	95.00 ± 0.00	0.110

^1^ Values in each row with different superscripts are significantly different (*p* < 0.05). For diet information refer to Table 2. ^2^ IBW = Initial body weight (g); FBI = Final body weight (g); WG = Weight gain (%); SGR = Specific growth rate (%/day); FE = Feed efficiency (%); PER = Protein efficiency ratio; SR = Survival (%).

**Table 4:** Whole-body proximate composition of the whiteleg shrimp (wet weight bases) fed the six experimental dies for eight weeks (Mean ± SD of triplicate observations) ^1^.

	Diets						*p*-Value
	CON	YH_0.5_	YH_1_	YH_2_	YH_4_	FM_25_	
Moisture	75.83 ± 0.42	76.07 ± 0.44	75.82 ± 0.17	75.78 ± 0.41	76.01 ± 0.58	76.06 ± 0.51	0.959
Crude protein	18.56 ± 0.06	18.58 ± 0.03	18.53 ± 0.06	18.53 ± 0.01	18.54 ± 0.02	18.55 ± 0.02	0.349
Crude lipid	1.29 ± 0.05	1.31 ± 0.04	1.35 ± 0.05	1.30 ± 0.06	1.32 ± 0.05	1.30 ± 0.03	0.827
Crude ash	3.43 ± 0.02	3.44 ± 0.04	3.42 ± 0.02	3.40 ± 0.01	3.45 ± 0.03	3.39 ± 0.02	0.848

^1^ Values in each row with different superscripts are significantly different (*p* < 0.05). For diet information refer to Table 2.

**Table 5:** Hemolymph biochemical parameters of the whiteleg shrimp fed the six experimental diets for eight weeks (Mean ± SD of triplicate observations) ^1^.

	Diets						*p*-Value
	CON	YH_0.5_	YH_1_	YH_2_	YH_4_	FM_25_	
AST ^2^	10.67 ± 2.08	12.67 ± 3.79	13.67 ± 2.89	11.67 ± 2.52	11.33 ± 3.21	9.33 ± 1.15	0.502
ALT ^3^	9.00 ± 1.73	9.67 ± 1.53	9.67 ± 1.53	10.33 ± 2.08	9.33 ± 0.58	9.00 ± 1.00	0.876
GLU ^4^	57.33 ± 11.15	61.33 ± 15.01	57.67 ± 8.14	58.67 ± 9.02	55.67 ± 8.74	53.33 ± 7.09	0.951

^1^ Values in each row with different superscripts are significantly different (*p* < 0.05). For diet information refer to Table 2. ^2^ Aspartate aminotransferase (U/L). ^3^ Alanine aminotransferase (U/L). ^4^ Glucose (mg/dL).

**Table 6:** Non-specific immune responses of the whiteleg shrimp fed the six experimental diets for eight weeks (Mean ± SD of triplicate observations) ^1^.

	Diets						*p*-Value
	CON	YH_0.5_	YH_1_	YH_2_	YH_4_	FM_25_	
SOD ^2^	43.60 ± 7.96	34.37 ± 5.88	39.66 ± 4.64	46.85 ± 4.67	45.03 ± 0.94	43.38 ± 4.34	0.261
LyZ ^3^	0.082 ± 0.01	0.076 ± 0.01	0.073 ± 0.03	0.080 ± 0.03	0.073 ± 0.03	0.077 ± 0.03	0.998

^1^ Values in each row with different superscripts are significantly different (*p* < 0.05). For diet information refer to Table 2. ^2^ Superoxide dismutase activity (% inhibition). ^3^ Lysozyme activity (U/mL).

**Table 7:** Intestinal morphology of whiteleg shrimp fed the experimental diets for 8 weeks (Mean ± SD of triplicate observations) ^1^.

	Diets						*p*-Value
	CON	YH_0.5_	YH_1_	YH_2_	YH_4_	FM_25_	
VL ^2^	33.80 ± 1.12 ^bc^	29.80 ± 2.10 ^c^	36.48 ± 2.33 ^b^	30.05 ± 1.16 ^c^	55.43 ± 4.79 ^a^	54.08 ± 1.34 ^a^	0.000
MLT ^3^	47.02 ± 1.50 ^c^	49.96 ± 2.26 ^c^	50.35 ± 1.33 ^c^	50.62 ± 1.24 ^c^	59.35 ± 2.37 ^b^	65.03 ± 3.73 ^a^	0.000

^1^ Values in each row with different superscripts are significantly different (*p* < 0.05). For diet information refer to Table 2. ^2^ Villi length (μm). ^3^ Muscular layer thickness (μm).

## Data Availability

The data that support the findings of this study are available on request from the corresponding author. The data are not publicly available due to privacy or ethical restrictions.
